# Therapeutic Effectiveness of Microneedling Radio Frequency in Different Areas of Periorbital Static Wrinkles: A Self‐Controlled Study

**DOI:** 10.1111/jocd.16645

**Published:** 2024-11-26

**Authors:** Feiyan Lin, Jinru Song, Ying Hua, Liangli Pan, Yao Guo, Gang Hu, Bin Yang

**Affiliations:** ^1^ Dermatology Hospital Southern Medical University Guangzhou Guangdong China

**Keywords:** Antera 3D, microneedling radio frequency, periocular static wrinkles

## Abstract

**Background:**

Periocular static wrinkles, which are common cosmetic concerns, lack an established effective treatment. Microneedling radio frequency (MNRF) has shown promise in skin rejuvenation; however, there is limited objective information on its long‐term effectiveness with regard to periocular static wrinkles.

**Aims:**

This study aimed to evaluate the clinical efficacy and safety of MNRF treatment for periocular static wrinkles.

**Method:**

Eighteen participants with moderate to severe wrinkles were enrolled in this study. MNRF treatment was applied to the periocular skin using MicroRF9 microneedles, which have a depth of 0.5–1.5 mm and a pulse width of 200–300 ms. MNRF treatment was administered twice with a 1‐month interval, and the participants were followed up for 6 months. The evaluation included four areas, namely the outer canthi, lower eyelid, inner canthi, and upper eyelid, by using clinical assessments and Antera 3D measurements by digitalized images and grading by clinicians.

**Results:**

The results showed significant improvement in all four areas assessed. Participants expressed high satisfaction with the treatment, and any adverse events, such as mild pain and redness, were temporary and resolved within a week.

**Conclusion:**

These findings confirm that MNRF is a safe and effective method for reducing periocular static wrinkles.

## 1 Introduction

The skin regions around the eyes are particularly susceptible to experiencing endogenic age‐related and exogenous photo‐related changes because of the thinner thickness, fewer oil glands, and exposure to long‐term and repetitive muscular contractions [1]. Periocular static wrinkles, a common skin alteration, are one of the predominant and visible phenotypes of skin aging that could deeply impact individuals' appearance [2].

Patients' desire to improve periocular static wrinkles are gradually increasing, such that various therapeutic technologies, including surgery, laser devices, and botulinum toxin and filler injections, have been developed [3, 4, 5, 6]. However, improving this area is still a challenge for clinicians because of the irregular shape and anatomical position that is close to the eyes. Therefore, it is necessary to develop a safe and effective method to improve periocular static wrinkles. Microneedling radio frequency (MNRF), a minimally invasive treatment, is one of the most frequently used therapies in the field of skin rejuvenation [7]. The microneedles deliver energy directly to produce thermal damage, which can induce collagen and adipose remodeling. The penetration depth of the microneedle system can be adjusted to prevent injury of the epidermis. MNRF can be used in various skin types with numerous dermatological indications, such as scars, melasma, hair thinning, skin laxity, acne vulgaris, and rosacea [5, 8, 9, 10, 11]. However, use of MNRF for improving periocular static wrinkles is rarely reported, and so far, few publications regarding an objective evaluation of periocular static wrinkles after long‐term application of MNRF are available.

This study aimed to evaluate the safety and efficacy of an MNRF system of negative pressure microneedle 9 (MicroRF9) microneedles for improving periocular static wrinkles by assessing clinical manifestations and measuring the wrinkle index with the Antera three‐dimensional (3D) image capture system.

## 2 Methods

### 2.1 Ethics Statement

This study was approved by the Dermatology Hospital of Southern Medical University Institutional Review Board (2021014), and informed consent was obtained from all patients.

### 2.2 Study Design and Population

This was a prospective, intraindividual‐controlled, single‐center study. Subjects aged 40–63 years were recruited in this study. Inclusion criteria were as follows: age between 30 and 70 years, female sex, presence of static wrinkles around the eyes, with a score of 2 or higher (Fitzpatrick Grade II or higher) for each site (including the outer corner of the eye, lower eyelid, inner canthi, and upper eyelid within the periorbital area).

The exclusion criteria were pregnancy, history of pathological scars, infection at the investigational treatment site, hypersensitivity to lidocaine, aesthetic treatments within the past 12 months such as laser skin resurfacing, dermabrasion, filler injections and botulinum toxin injection, and so on.

### 2.3 Materials

**FIGURE 1:**
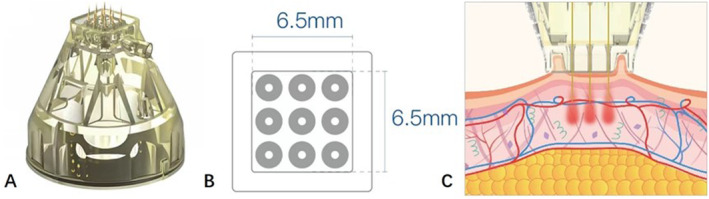
The structure and model figures of MicroRF9. (A) MicroRF9 handpiece consisting of nine minimally invasive noninsulated microneedle electrodes. (B) The total contact area of MicroRF9 handpiece is 1 cm^2^, and the inner area of the negative pressure tank is 0.47 cm^2^. (C) Schematic diagram of negative pressure suction.

An invasive, bipolar, pulsed‐type, alternating current, radio frequency device with noninsulated needles MNRF device (Model: United, Shenzhen Peninsula Medical Co. Ltd.) was used in this study. This device, MicroRF9, has a handpiece with a disposable single‐use tip consisting of nine minimally invasive noninsulated microneedle electrodes or pins (Figure 1A). The pins are arranged in a 3 × 3 pattern (diameter: 0.3 mm, distance between electrodes: 2.5 mm, Figure 1B). They have a tip length of 3.0 mm, and their penetration depth can be adjusted from 0.5 to 3 mm. During the treatment process, the negative pressure suction provided by the MicroRF9 reduces the patient's discomfort and increases the safety of the operation (Figure 1C).

### 2.4 Treatment Procedure

Before treatment, 10 g topical anesthetic lidocaine cream was applied to the participant's periocular area for 1 h. After removing the lidocaine cream, the MNRF device was applied to the periocular skin. During treatment, the pulse width of the device was 200–300 ms, generating a considerable power at 4.0–10.0 W and directly delivering energy using MicroRF9 microneedles with a 0.5–1.5‐mm depth. After 10–15 mins of treatment, immediate cooling of the skin was provided with saline gauze for 15 min. No other special instructions or skincare products were given. The treatment was repeated once after a 1‐month period.

### 2.5 Physician Subjective Score

**FIGURE 2:**
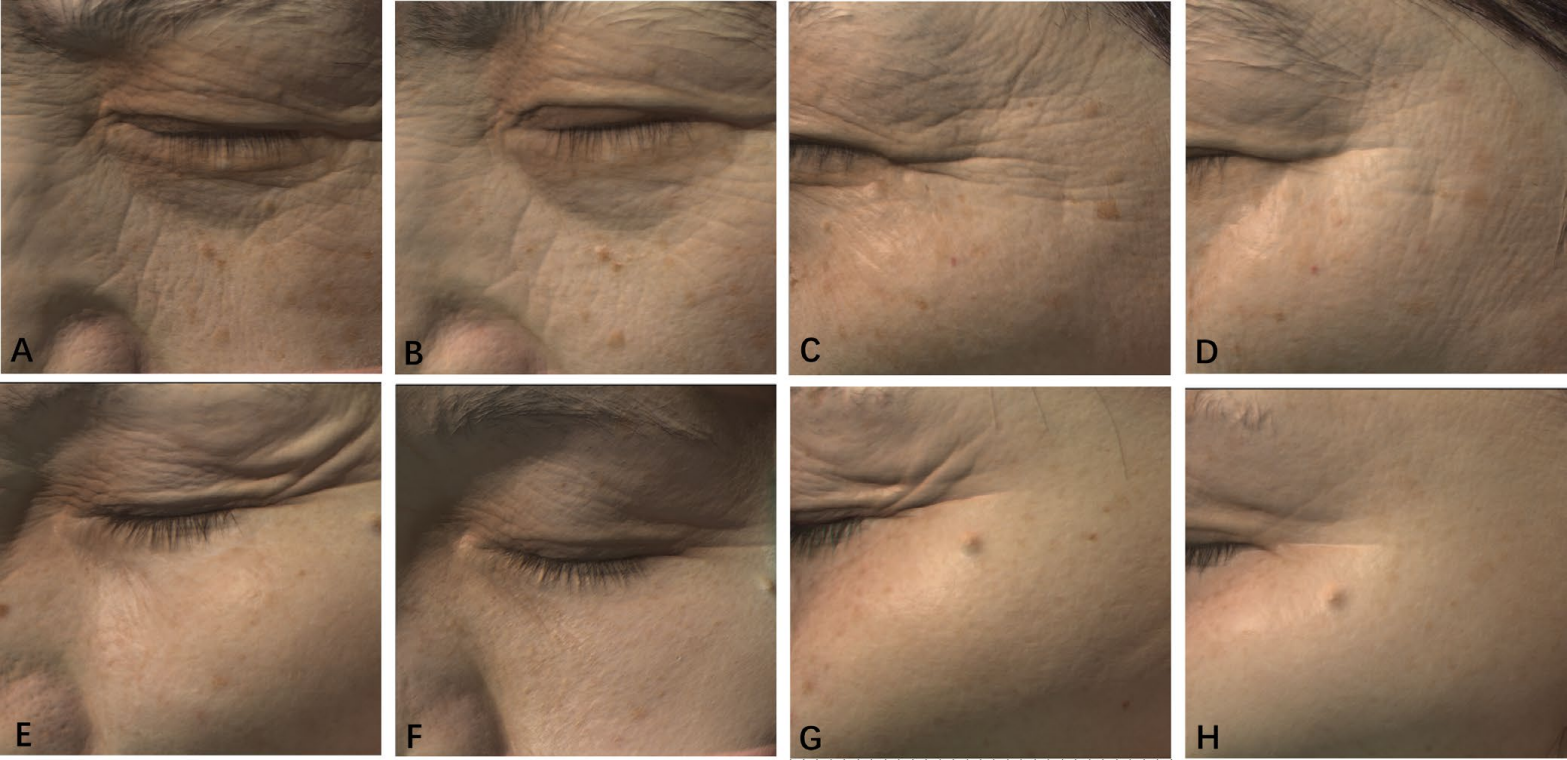
Clinical photographs of two female patients before and 6 months after treatment in different angles. (A, C) The clinical figure of subject 1 at baseline. (B, D) The clinical figure of subject 1 treated by MNRF at 6 months after treatment. (E, G) The clinical figure of subject 2 at baseline. (F, H) The clinical figure of subject 2 treated by MNRF at 6 months after treatment.

Photo‐documentation was gathered with the VISIA‐CR (Canfield Scientific Inc., Fairfield, NJ, USA). Two independent dermatologists who were not involved in this trial assessed wrinkles at baseline and 6 months after the last treatment based on the Fitzpatrick Wrinkle and Elastosis Scale (FWES) [12], which is shown in Table 1. The FWES was used for assessing treatment efficacy over the four areas, namely the outer canthi, lower eyelid, inner canthi, and upper eyelid at the 6‐month follow‐up with the baseline as reference (Figure 2).

**TABLE 1:** Fitzpatrick Wrinkle and Elastosis Scale.

Class	Wrinkling	Score	Degree of elastosis
I	Fine wrinkles (rhytides)	1–3	Mild (fine textural changes with subtly accentuated skin lines)
II	Fine to moderate depth wrinkles, moderate number of lines	4–6	Moderate (distinct papular elastosis [individual papules with yellow translucency under direct lighting] and dyschromia)
III	Fine to deep wrinkles, numerous lines, with or without redundant skin folds	7–9	Severe (multipapular and confluent elastosis [thickened yellow and pallid] approaching or consistent with cutis)

### 2.6 Measurement of the Wrinkle Index

The Antera 3D image capture system (ANTERA 3D, Miravex Limited, Dublin, Ireland) is an objective device, containing a camera for image acquisition and corresponding software for analysis of the skin [13]. The objective evaluations included measurement of the maximum depth and indentations of the wrinkles over the four areas of outer canthi, lower eyelid, inner canthi, and upper eyelid. These data were measured at baseline and the 6‐month follow‐up by this equipment.

### 2.7 Patient Subjective Satisfaction

The five‐point Global Aesthetic Improvement Scale (GAIS) questionnaire was used to evaluate satisfaction with the treatment outcomes of participants [14]. The scores range of −1 to 3, with −1 = worse than the baseline, 0 = no change, 1 = improved, 2 = much improved, and 3 = very much improved.

### 2.8 Adverse Effects

Furthermore, any adverse effects such as erythema, edema, pain, postoperative bruising, and bleeding were recorded [13]. And the duration of these adverse effects also be followed up.

### 2.9 Statistical Analyses

We have evaluated both sides of bilateral periocular static wrinkles by the wrinkles grading scores and Antera 3D image capture system and counted the average scores. And the results were analyzed using the paired chi‐squared test. SPSS, version 24.0 (IBM Corp., Armonk, NY, USA) was used to perform the statistical analysis, and *p*‐values < 0.05 were considered statistically significant.

## 3 Results

### 3.1 Therapeutic Outcomes

As shown in Table 1, 18 women aged between 40 and 63 years with moderate to severe Fitzpatrick wrinkles participated in this study and completed two sessions of treatments and follow‐up at 6 months. Table 2 shows the effect of treatment at the four different areas of evaluation by FWES. There were mean improvements in the Fitzpatrick Wrinkle Scale score of 2.21, 1.62, 4.6, and 1.03 at the outer canthi, lower eyelid, inner canthi, and upper eyelid, respectively, at baseline compared with 6 months after treatment (all, *p* < 0.001). These results show significant improvement of wrinkles after treatment compared with baseline.

**TABLE 2:** Clinical improvement according to the Fitzpatrick Wrinkle Scale scores at baseline and after the 6‐month follow‐up.

	Baseline	6 months after treatment	Average score reduction	*p*
Outer canthi	5.68 ± 2.35	3.47 ± 1.39	2.21	0.000
Lower eyelid	6.44 ± 1.97	4.82 ± 1.66	1.62	0.000
Inner canthi	6.41 ± 1.83	1.81 ± 1.65	4.6	0.000
Upper eyelid	5.56 ± 2.40	4.53 ± 2.00	1.03	0.004

### 3.2 Maximum Depths and Indentations of the Wrinkles

**FIGURE 3:**
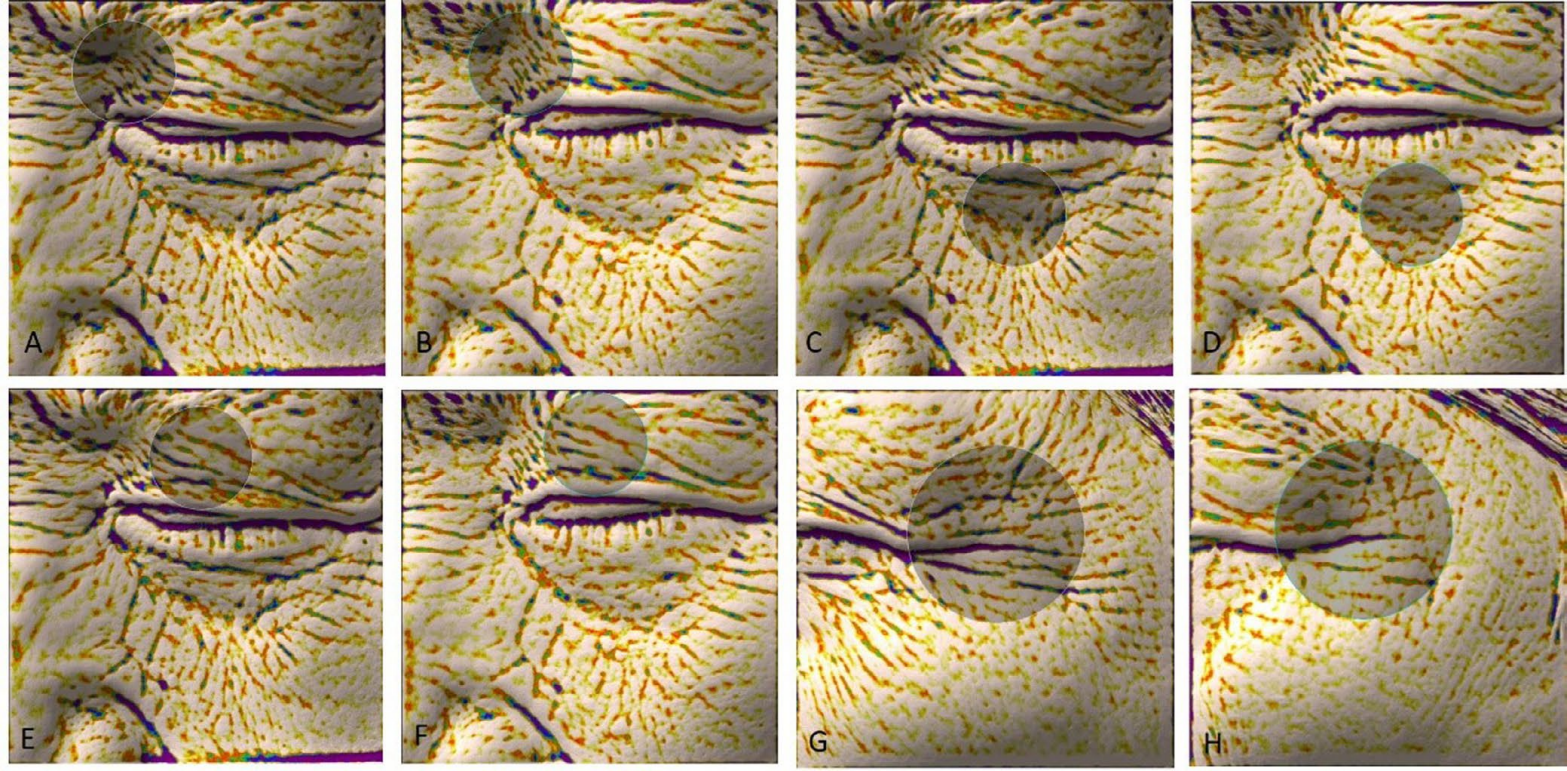
Representative photographs of the Antera 3D images captured in different positions before treatment and 6 months after treatment. (A) The image of inner canthi at baseline. (B) The image of inner canthi at 6 months after treatment. (C) The image of lower eyelid at baseline. (D) The image of lower eyelid at 6 months after treatment. (E) The image of upper eyelid at baseline. (F) The image of upper eyelid at 6 months after treatment. (G) The image of outer canthi at baseline. (H) The image of outer canthi at 6 months after treatment.

The maximum depths and indentations of wrinkles at the four areas of interest were measured by the Antera 3D image capture system. As shown in Figure 3 and Table 3, significant differences in the maximum depths were observed between before and after treatment at all sites. The maximum depths were significantly decreased at 6 months after treatment in the outer canthi (0.160 ± 0.054 vs. 0.136 ± 0.047, *p* < 0.01), lower eyelid (0.112 ± 0.037 vs. 0.078 ± 0.025, *p* < 0.001), inner canthi (0.134 ± 0.058 vs. 0.094 ± 0.031, *p* < 0.001), and upper eyelid (0.119 ± 0.048 vs. 0.101 ± 0.041, *p* = 0.008) of the periorbital area compared with pretreatment values.

**TABLE 3:** Maximum depths and indentations according to the Antera 3D image capture system.

Point	3D measurement	Baseline	After treatment	*p*
Outer canthi	The maximum depths	0.160 ± 0.054	0.136 ± 0.047	0.01
	The volume	10.74 ± 3.33	9.54 ± 2.57	0.007
Lower eyelid	The maximum depths	0.112 ± 0.037	0.078 ± 0.025	0.0001
	The volume	12.03 ± 3.43	10.06 ± 2.76	0.004
Inner canthi	The maximum depths	0.134 ± 0.058	0.094 ± 0.031	0.001
	The volume	13.60 ± 3.49	11.40 ± 2.71	0.0001
Upper eyelid	The maximum depths	0.119 ± 0.048	0.101 ± 0.041	0.008
	The volume	13.71 ± 4.02	12.66 ± 4.43	0.015

The indentations were also significantly decreased at 6 months after treatment in the outer canthi (10.74 ± 3.33 vs. 9.54 ± 2.57, *p* = 0.007), lower eyelid (12.03 ± 3.43 vs. 10.06 ± 2.76, *p <* 0.0001), inner canthi (13.60 ± 3.49 vs. 11.40 ± 2.71, *p* < 0.0001), and upper eyelid (13.71 ± 4.02 vs. 12.66 ± 4.43, *p* = 0.015) of the periorbital area compared with pretreatment values.

### 3.3 Adverse Effects

All patients experienced pain during FMR, four patients (22.2%) reported severe pain, six patients (33.3%) reported moderate pain, eight patients reported mild pain (44.4%), and no patients reported no pain. After surgery, patients developed erythema, edema, and pain for average durations of 68.82 h, 49.05 h, and 43.84 h, respectively. The incidence of postoperative bruising was 5.88%, with an average duration of 5.5 days for resolution. The incidence of postoperative bleeding was 29.41%, with an average duration of 0.71 h.

### 3.4 Patient Satisfaction

Overall, 11.1% of patients reported being very much improved, 55.6% reported being much improved, 33.3% reported improvement, and 0% reported worse outcomes than the baseline or no change.

## 4 Discussion

This study aimed to explore the safety and efficacy of MNRF for improving periocular static wrinkles. Our results demonstrated clinical improvement, as evaluated by clinicians, and decreases in the maximum depths and indentations, according to the Antera 3D image capture system, which can evaluate the skin profilometry of a prespecified target by means of computer analysis [15, 16]. These data suggest that MNRF could improve the wrinkles in four areas, including the outer canthi, lower eyelid, inner canthi, and upper eyelid. Additionally, our results indicate that this technology results in a significantly continuous improvement of periocular wrinkles at least at 6 months after treatment compared with baseline.

The mechanism of MNRF for wrinkles may be related to the immediate tissue contraction caused by the cleavage of hydrogen bonds in the triple helix structure of collagen, which thickens and shortens collagen fibril. Delayed tissue contraction is caused by inflammation, which triggers a cascade of wound healing that results in 3–4 months of angiogenesis [17], new collagen production [18], and elastic recombination [19].

Some adverse effects such as mild pain, erythema, and edema were observed, although these issues resolved within 3 days. Postprocedural treatment such as immediate cooling of the skin should be performed to relieve patient discomfort. Patients should also apply topical moisturizing agents to moisturize and repair the skin during the postprocedural period. With these standard postprocedures, patients had a low risk of adverse effects. Moreover, we used a MicroRF9 needle head. In comparison with traditional tip, the distinguishing feature of this tip is its negative pressure suction capability, which allows the eyelid skin to be lifted away from the eyeball for needle insertion through negative pressure attraction. This feature improves patient comfort during treatment and reduces thermal damage to the subcutaneous tissues of the eyelid by minimizing heat dissipation. In addition, MNRF can be safely used in all Fitzpatrick skin types because it is not chromophore‐dependent.

This study has some limitations, including a small sample size, the absence of evaluations between each treatment session and the lack of comparison with other techniques which limits the ability to compare MNRF directly with other treatments. Additionally, more studies on the molecular mechanism and randomized controlled trials comparing MNRF with other treatments are needed.

## 5 Conclusion

This study's results confirm that MNRF is safe and effective for improving periocular static wrinkles.

## Author Contributions

B.Y. and G.H. contributed to the conception of the study. F.L. and J.S. contributed significantly to analysis and manuscript preparation. J.S. performed the data analyses and wrote the manuscript. Y.H. and L.P. helped collect the data and perform treatment. Y.G. helped recruit the patients. All authors read and approved the final manuscript.

## Consent

Informed consent was obtained from all patients.

## Conflicts of Interest

The authors declare no conflicts of interest.

## Data Availability

The datasets generated and analyzed during the current study are available from the corresponding author on reasonable request.
